# Diagnosing Mitochondrial Encephalopathy, Lactic Acidosis, and Stroke-Like Episodes (MELAS) Syndrome in a Young Adult Female Patient With Seizures and Lactic Acidosis

**DOI:** 10.7759/cureus.88031

**Published:** 2025-07-15

**Authors:** Ishan A Sane, Jessica R Gupte

**Affiliations:** 1 Internal Medicine, Smt. Kashibai Navale Medical College and General Hospital, Pune, IND

**Keywords:** encephalomyopathy, lactic acidosis, melas syndrome, mitochondrial disorder, red ragged fibers, stroke-like episodes

## Abstract

The aim of this case report is to highlight the diagnostic challenges and clinical presentation of mitochondrial encephalopathy, lactic acidosis, and stroke-like episodes (MELAS) syndrome, which is a rare, maternally inherited mitochondrial disorder. MELAS typically manifests with a constellation of neurological and systemic symptoms, including seizures, lactic acidosis, stroke-like episodes, and progressive cognitive decline. Mutations in mitochondrial DNA impair oxidative phosphorylation and result in widespread cellular dysfunction.

We report the case of a 33-year-old female patient who presented with seizures, altered mental status, and focal neurological deficits. Laboratory evaluation revealed elevated serum lactate, and neuroimaging demonstrated stroke-like lesions not confined to vascular territories. A muscle biopsy showed abnormal mitochondrial accumulation, and electron microscopy detected ragged red fibers, which is confirmatory of mitochondrial cytopathy. The patient was managed symptomatically in the intensive care unit with antiepileptics, corticosteroids, and a mitochondrial cocktail comprising coenzyme Q10, L-arginine, L-carnitine, and B-complex vitamins. Plasmapheresis was also performed during initial management due to diagnostic uncertainty. The patient showed gradual clinical improvement and was discharged on supportive therapy.

This case emphasizes the importance of early recognition of atypical stroke-like presentations and metabolic derangements in young patients. MELAS syndrome should be considered in the differential diagnosis of stroke mimics, especially in the absence of vascular risk factors. Timely diagnosis, supportive care, and long-term follow-up, including genetic counselling, are essential for optimizing outcomes in these patients.

## Introduction

Mitochondrial encephalopathy, lactic acidosis, and stroke-like episodes (MELAS) syndrome is a degenerative condition resulting from mitochondrial dysfunction. Approximately 80% of individuals with MELAS possess a mutation in mitochondrial DNA (mtDNA). This disorder is marked by normal development in early life, followed by the onset of specific symptoms, usually occurring before the age of 40 years [1]. It is an extremely rare genetic mitochondrial disease with a prevalence of 16-18 per 100,000 persons [2].

MELAS syndrome is a rare disorder with significant genetic implications, involving mutations in the mitochondria in the MT-ND1, MT-ND5, MT-TH, MT-TL1, MT-TV, etc., genes, and it is inherited maternally. This condition was first identified by Pavlakis et al. in 1984 [3,4].

In most patients, the onset of the disease occurs early and is considered typical. The condition worsens over time, leading to more pronounced symptoms. Common signs include learning difficulties, a decline in cognitive abilities, intolerance to physical activity, and weakness in the limbs. Seizures frequently occur during stroke episodes. Patients often experience episodic migraine headaches accompanied by nausea and vomiting, which typically precede the stroke-like events. Ultimately, all patients experience cognitive decline [5].

This condition's clinical diagnosis is determined by a number of factors, such as imaging results, genetic variation analysis, clinical symptoms, and, in certain situations, muscle biopsy. Serum biochemical changes are characteristic of acute attacks, and cortical infarcts with restricted diffusion that are not associated with any particular vascular territory are revealed by distinctive magnetic resonance imaging (MRI) findings. Mitochondrial genetic testing is typically necessary to confirm the diagnosis [6].

There is currently no standardized treatment protocol for patients with MELAS syndrome. Management is primarily supportive and symptom-based, typically requiring a multidisciplinary approach [7].

## Case presentation

A 33-year-old female patient was brought to the emergency department with complaints of intermittent, bilateral diffuse headache and tingling sensations involving the entire body for the past two months. She also complained of involuntary movements of the right upper and lower limbs and generalized tonic-clonic seizures for the last five days. There was no relevant past medical history. The patient did not report any similar episodes in the past and had no comorbidities, addictions, known drug allergies, or prior use of oral contraceptives or anticoagulants. There was also no personal or family history suggestive of stroke, transient ischemic attack, or autoimmune disease.

On arrival, her vitals were normal, and her Glasgow Coma Scale (GCS) score was 4/15, following which the patient was immediately intubated and started on mechanical ventilation. Neurological examination revealed increased muscle tone bilaterally, brisk deep tendon reflexes, and bilateral Babinski signs, indicating diffuse cortical involvement. Due to intubation and altered sensorium, cranial nerve evaluation, sensory examination, and cerebellar function tests could not be performed. There were no clinical signs of meningeal irritation.

The patient was started on intravenous (IV) levetiracetam 500 mg OD and oral carbamazepine 200 mg BD for seizure control. Empirical treatment with IV methylprednisolone 1 g was initiated in view of a suspected inflammatory etiology. Dual antiplatelet therapy (aspirin 75 mg OD and clopidogrel 75 mg OD) was also started empirically, considering the possibility of a vascular infarct. The patient was admitted to the intensive care unit (ICU) for further management and evaluation.

**Figure 1:**
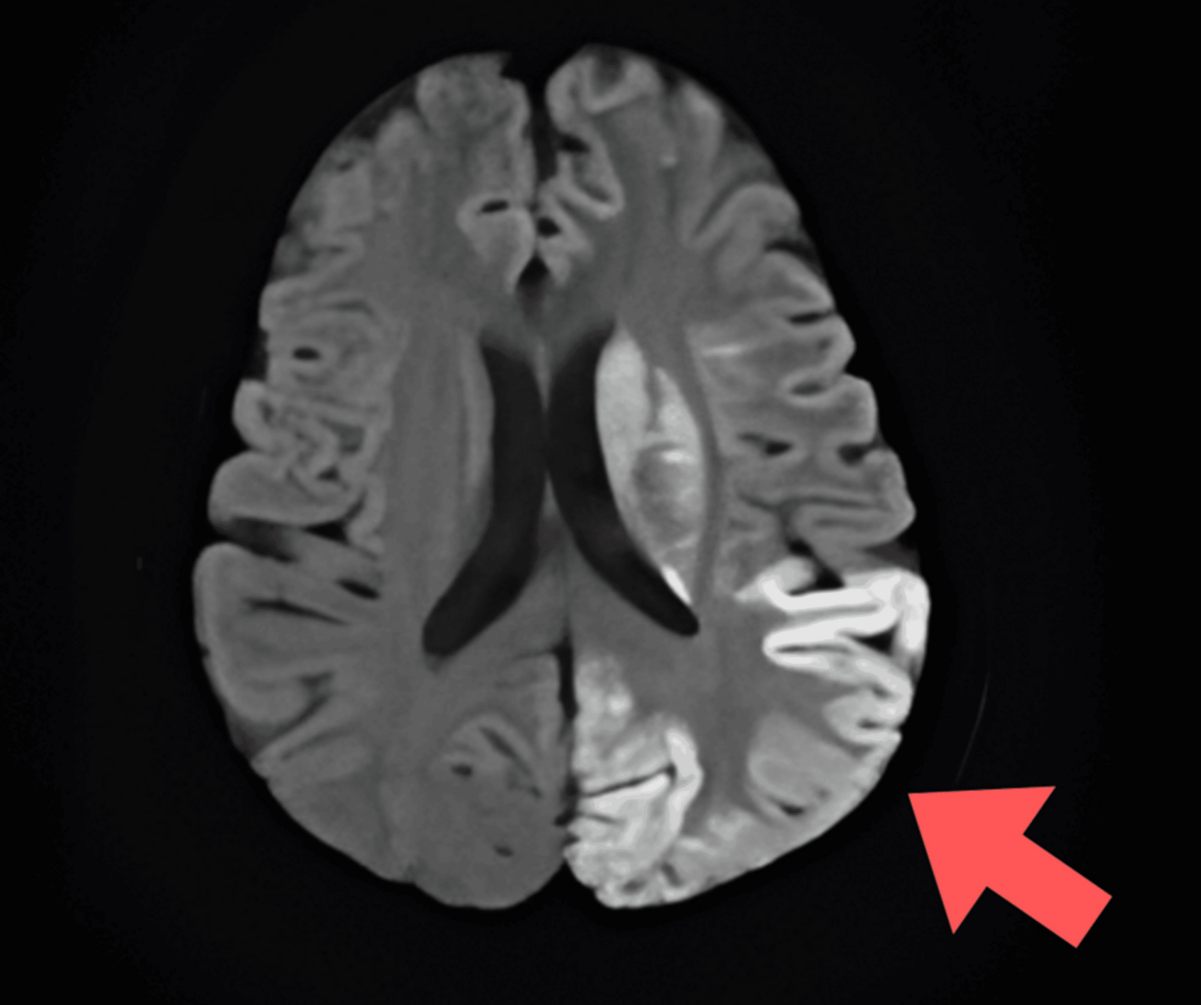
Diffusion-weighted magnetic resonance imaging (DW-MRI) showing hyperintensity in the right parieto-occipital region.

**Figure 2:**
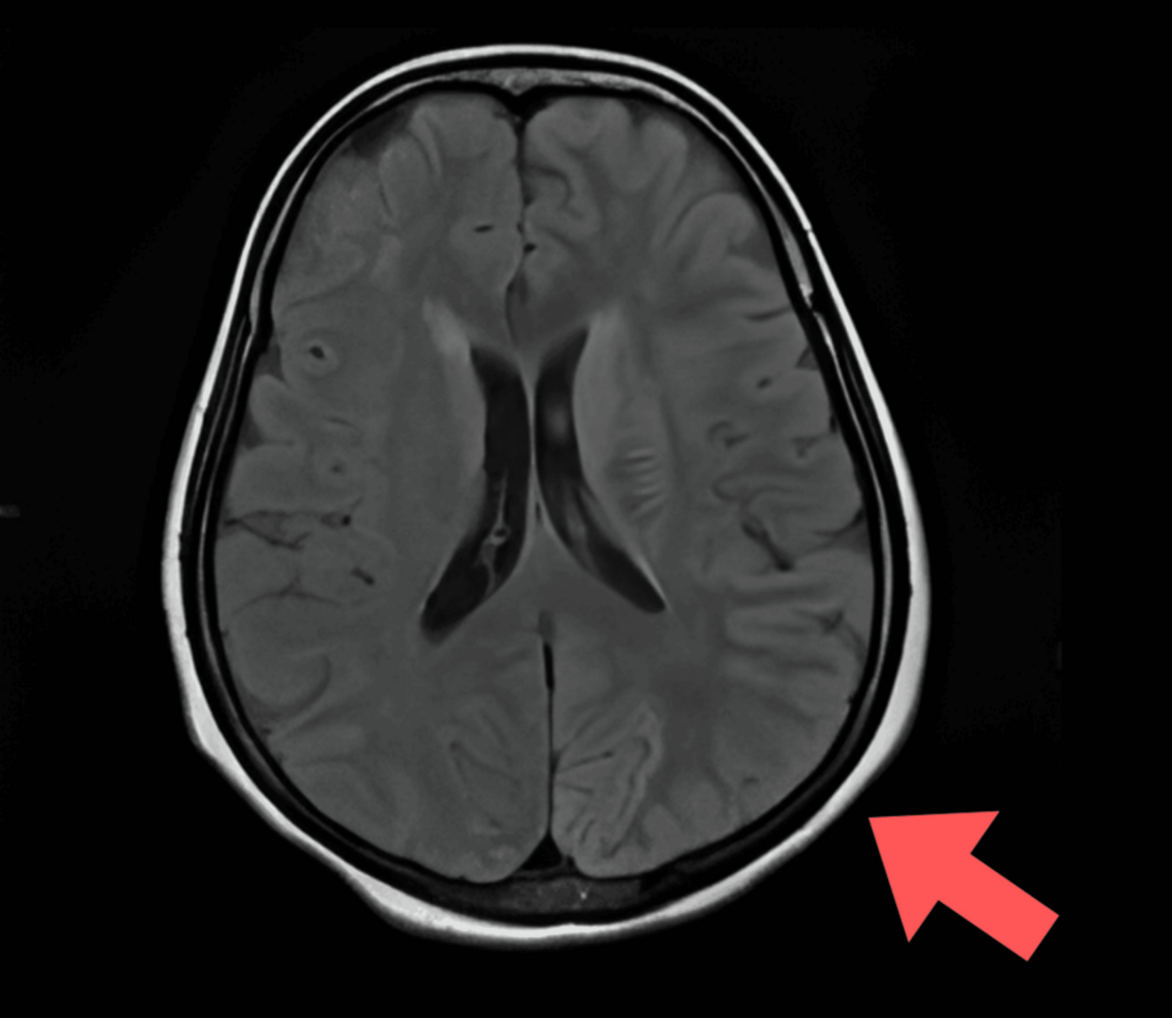
Fluid-attenuated inversion recovery (FLAIR) imaging showing hyperintensity in the right parieto-occipital region.

**Figure 3:**
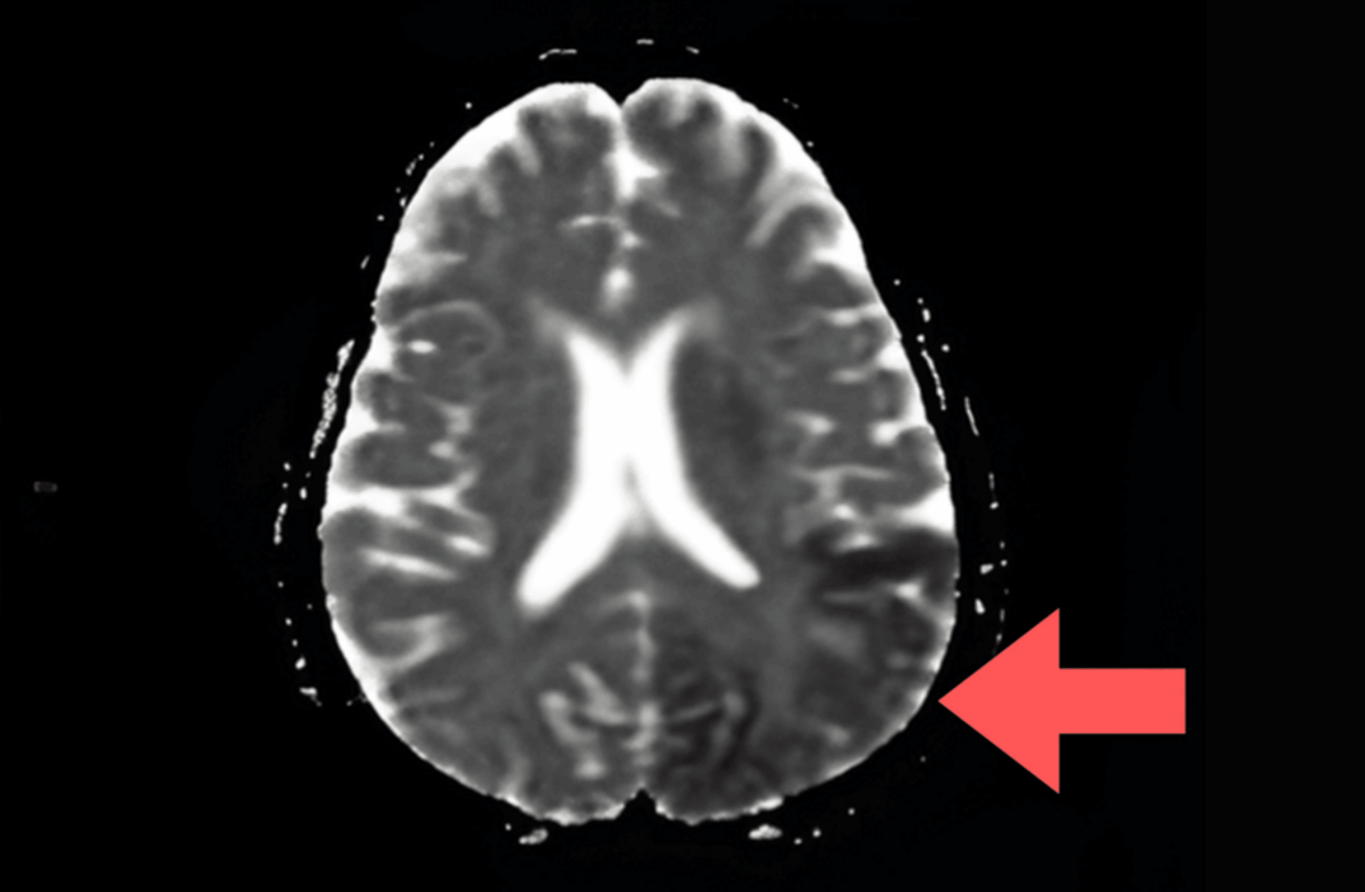
Apparent diffusion coefficient (ADC) map showing mild isointensity in the right parieto-occipital region.

Laboratory investigations were normal except that the patient had elevated serum lactate levels (31.6 mg/dL), indicating lactic acidosis. Diffusion-weighted MRI of the brain showed hyperintense areas and acute infarcts in the left high parietal region, left corona radiata, and left frontoparietal and temporo-occipital lobes. Fluid-attenuated inversion recovery (FLAIR) imaging demonstrated hyperintensity in the left parietal region, while apparent diffusion coefficient (ADC) images showed isointensity in the bilateral thalami. The radiological appearance of regions that were hyperintense on diffusion-weighted imaging (DWI) and FLAIR but isointense on ADC suggested subacute infarcts. These findings are not typical of a vascular infarct and instead point toward a metabolic stroke-like lesion (Figures 1-3).

**Figure 4:**
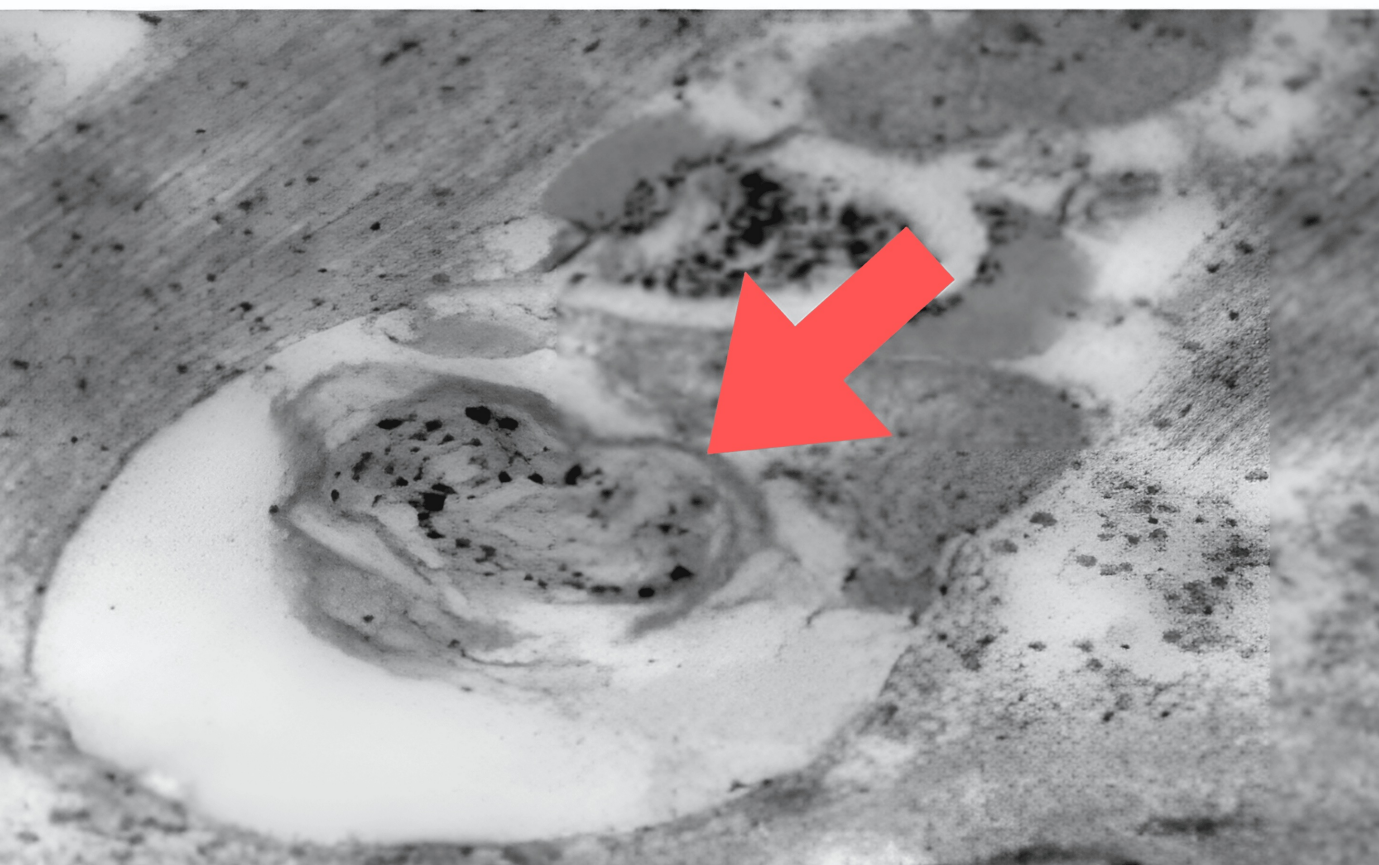
Electron microscopy of muscle tissue showing ragged red fibers.

A muscle biopsy was performed, which on light microscopy revealed mild variation in muscle fiber size without any evidence of myocyte necrosis, internalized nuclei, degenerative changes, or perivascular inflammation. These findings effectively ruled out inflammatory myopathies such as dermatomyositis or polymyositis. Electron microscopy of the muscle tissue revealed the presence of ragged red fibers (RRFs), which are abnormal, enlarged mitochondria located subsarcolemmally (Figure 4). These findings are pathognomonic for mitochondrial cytopathies. These histopathological findings, in conjunction with the clinical presentation, lactic acidosis, and neuroimaging features, confirmed the diagnosis of MELAS syndrome. 

Supportive management was continued in the ICU. Nutritional support was provided via Ryle’s tube, and the patient was maintained on oxygen therapy. Plasmapheresis was performed in the early stage of management to reduce circulating toxic metabolites and inflammatory mediators, given the initial diagnostic uncertainty. IV steroids were continued initially in order to reduce cerebral edema. A mitochondrial cocktail therapy was initiated, comprising coenzyme Q10, L-arginine, L-carnitine, and B-complex vitamins including thiamine and riboflavin, aimed at enhancing mitochondrial function and improving cellular energy metabolism.

Over the following days, the patient's condition improved steadily. Her GCS score improved, she began regaining consciousness and cognitive function, and the frequency of seizures reduced. She was discharged in a stable condition on a tapering dose of corticosteroids, oral antiepileptic drugs, and nutritional mitochondrial supplements. She was advised to follow up regularly in the neurology outpatient clinic.

Neurorehabilitation and physiotherapy were recommended to assist in the recovery of neurological function. Genetic counselling was advised for the patient and her family members due to the hereditary nature of mitochondrial disorders. Long-term follow-up was emphasized, with the goal of monitoring neurological status, preventing complications, and optimizing quality of life.

## Discussion

In our case, the patient was managed symptomatically in the ICU with antiepileptics, corticosteroids, and mitochondrial supplements. The diagnosis of MELAS was based on clinical features, lactic acidosis, and atypical neuroimaging findings. Confirmation was achieved through muscle biopsy, which revealed RRFs on electron microscopy, consistent with mitochondrial cytopathy. Genetic testing of the patient was not done.

Mitochondrial cytopathies are typically categorized into three distinct clinical groups: MELAS, myoclonus epilepsy with RRFs (MERRF), and chronic progressive external ophthalmoplegia (CPEO) [8]. MELAS syndrome is a rare disorder inherited maternally, mainly due to mutations in mtDNA, particularly the m.3243 A>G mutation in the MT-TL1 gene. These mutations disrupt mitochondrial oxidative phosphorylation, resulting in systemic effects such as stroke-like episodes, lactic acidosis, seizures, muscle weakness, and progressive neurodegeneration [9].

Clinical stroke, RRFs, exercise intolerance, lactic acidosis, seizures, and symptom onset before the age of 40 were observed in over 90% of cases. Encephalopathy may present as mental retardation or dementia. Elevated lactate levels can be detected in serum and/or cerebrospinal fluid samples. Stroke-like episodes often involve symptoms and signs of hemianopia and hemiplegia [10].

Our patient exhibited encephalopathy characterized by seizures and reduced consciousness, along with increased serum lactate and stroke-like episodes with focal neurological deficits and cortical involvement. MRI findings are crucial for diagnosing and monitoring MELAS syndrome, as they reveal distinctive patterns. MRI is the preferred method for this purpose, showing global alterations in gray-white matter differentiation, multifocal cortical and subcortical lesions that span vascular territories, and varying levels of generalized cerebral and cerebellar atrophy. Diffusion-weighted MRI displays a high-intensity cortical ribbon-like signal indicative of diffusion restriction. During episodes, restricted diffusion can be observed in the cortex, subcortical white matter, and basal ganglia [11].

Both gray and white matter in the brain appear hyperintense on FLAIR. A DWI hyperintense signal with a corresponding decrease on ADC maps may be seen in the cortical regions of the lesion, indicating cytotoxic edema. The reduction in diffusibility is relatively mild compared to acute ischemia and likely reflects a state of diminished cellular energy [12].

In our patient, MRI findings showed hyperintensities on FLAIR affecting both gray and white matter, along with DWI hyperintensities and relatively mild isointense ADC changes. These characteristics are indicative of cytotoxic edema due to impaired mitochondrial energy metabolism, rather than acute vascular infarction, confirming a diagnosis of MELAS.

The recognition of RRFs as a significant histological marker of mitochondrial disorders was made before molecular diagnostic methods were developed. Usually, RRF can be identified via Gomori staining. RRF typically exhibits mitochondria with structural abnormalities, often containing paracrystalline inclusions visible under electron microscopy. These paracrystalline formations are considered highly indicative and are commonly found in RRF. They represent the crystallization of mitochondrial creatine kinase, which occurs due to increased activity as a compensatory response to energy shortages. The term ragged red fiber equivalents is used to describe muscle fibers with mitochondrial buildup [13].

Presently, there is no effective treatment for MELAS syndrome, so symptomatic management is employed. One of the most promising treatments for MELAS syndrome involves L-arginine, which can rapidly alleviate the condition by lowering lactate levels and enhancing microcirculation through increased nitric oxide production. Additionally, medications that facilitate electron transfer in the respiratory chain (such as coenzyme Q10, cytochrome C, and ascorbic acid), activate beta-oxidation (L-carnitine), and neutralize reactive oxygen species (lipoic acid) are also given to the patient [14].

Although current treatments for MELAS are mainly supportive, various therapeutic strategies have been explored with limited success. Genetic counselling plays a crucial role in managing MELAS patients. Emerging reproductive technologies may help reduce the recurrence of MELAS in future generations. Progress in gene therapy research offers hope for future treatments [15].

## Conclusions

MELAS syndrome is a rare mitochondrial disorder with a wide spectrum of clinical manifestations, often mimicking other neurological conditions and posing significant diagnostic challenges. In our case, a multidisciplinary approach and a high index of suspicion enabled timely recognition and diagnosis based on clinical presentation, neuroimaging, serum lactate levels, and confirmatory muscle biopsy with electron microscopy. Although no curative treatment currently exists, supportive management with antiepileptics, corticosteroids, and mitochondrial-targeted supplements led to clinical improvement. Early identification and intervention are essential to prevent disease progression and improve quality of life. Long-term follow-up, neurorehabilitation, and genetic counselling remain vital components of comprehensive care for MELAS patients.
